# Spezialisierte ambulante Palliativversorgung für Kinder, Jugendliche und ihre Familien – die besonderen Belange der Zielgruppe. Ergebnisse der ELSAH-Studie

**DOI:** 10.1007/s00103-022-03500-7

**Published:** 2022-02-02

**Authors:** Jennifer Engler, Dania Schütze, Michaela Hach, Cornelia Ploeger, Fabian Engler, ELSAH-Studiengruppe, Lisa-R. Ulrich, Hannah Seipp, Katrin Kuss, Stefan Bösner, Jörg Haasenritter, Ferdinand Gerlach, Antje Erler

**Affiliations:** 1 Institut für Allgemeinmedizin, Goethe-Universität Frankfurt am Main, Theodor-Stern-Kai 7, 60590 Frankfurt am Main, Deutschland; 2 Fachverband SAPV Hessen e. V., Weihergasse 15, 65203 Wiesbaden, Deutschland

**Keywords:** Spezialisierte ambulante Palliativversorgung, Kinder und Jugendliche, Pädiatrie, Versorgungsstrukturen, Lebenslimitierende Erkrankungen, Specialized outpatient palliative care, Children and adolescents, Pediatrics, Health care structures, Life-limiting conditions

## Abstract

**Hintergrund und Ziel:**

Lebenslimitierend erkrankte Kinder und Jugendliche mit komplexem Symptomgeschehen haben Anspruch auf eine spezialisierte ambulante Palliativversorgung (SAPV). In der Richtlinie zur SAPV heißt es lediglich: „Den besonderen Belangen von Kindern und Jugendlichen ist Rechnung zu tragen.“ Das Ziel der Studie ist es deshalb, diese besonderen Belange zu identifizieren und Empfehlungen zur Überarbeitung der SAPV-Richtlinie zu formulieren.

**Methoden:**

Sequenzielles Mixed-Methods-Design mit Fragebogenerhebungen, qualitativen Interviews, teilnehmenden Beobachtungen und Fokusgruppendiskussionen mit Angehörigen, Patient*innen und Leistungserbringer*innen der SAPV in Hessen sowie der Auswertung von Dokumentationsdaten der hessischen SAPV-Teams.

**Ergebnisse:**

Kinder und Jugendliche in der SAPV leiden an komplexen, oftmals seltenen Erkrankungen und bedürfen einer besonders aufwendigen Palliativversorgung durch ein Team mit pädiatrischer Expertise. Die SAPV muss die gesamte Familie einbeziehen und oftmals überregional verteilte Versorger*innen koordinieren. Zudem ist eine besonders aufwendige psychosoziale Versorgung von Patient*innen und Angehörigen notwendig. Die SAPV für Kinder und Jugendliche ist weniger bekannt als die SAPV für Erwachsene und der Zugang für die Familien deshalb oft schwierig. Für lebenslimitierend erkrankte Kinder und Jugendliche, die zwar einer aufsuchenden Palliativversorgung bedürfen, jedoch keinen Bedarf an einer so intensiven Betreuung wie in der SAPV haben, besteht eine Versorgungslücke.

**Fazit:**

Die SAPV von Kindern und Jugendlichen sowie von volljährigen Patient*innen, die seit dem Kindes- und Jugendalter erkrankt sind, bedarf einer eigenständigen Versorgungsform mit Vergütungsmodalitäten, die den besonderen Versorgungsbedarf und -aufwand abbilden.

## Abstract (en)

**Background and aim:**

Children and adolescents with life-limiting conditions and complex symptoms are eligible for specialized outpatient palliative care (SOPC). The SOPC guideline in Germany solely states: “The special needs of children and adolescents shall be considered.” This study aims to identify these special needs and to develop recommendations for a revision of the SOPC guideline.

**Methods:**

We used a sequential mixed-methods design including surveys, qualitative interviews, participant observations, and focus group discussions with relatives, patients, and team members of the SOPC in Hesse, Germany. Furthermore, we analyzed documentation data of the Hessian SOPC teams.

**Results:**

Children and adolescents in SOPC suffer from complex and often rare diseases. They need elaborate palliative care delivered by a team with pediatric expertise. SOPC must include the whole family and coordinate healthcare providers that are stretched regionally. Furthermore, patients and relatives need elaborate psychosocial care. SOPC for children and adolescents is less well-known than SOPC for adults, and access for families is often difficult. There is a healthcare gap for children and adolescents with life-limiting diseases who need palliative care at home but not of the intensive kind provided by SOPC.

**Conclusions:**

SOPC for children, adolescents, and adults who have been diseased since their childhood and adolescence must be delivered within an independent structure, including a reimbursement scheme that takes the special care efforts for this patient group into consideration.

## Hintergrund

Seit 2007 haben Menschen mit einer nicht heilbaren, fortschreitenden und weit fortgeschrittenen Erkrankung, die ihre Lebenserwartung verkürzt und die eine besonders aufwendige Versorgung benötigen, Anspruch auf spezialisierte ambulante Palliativversorgung (SAPV) in ihrer vertrauten häuslichen oder familiären Umgebung [1]. Ziel von SAPV ist es, „die Lebensqualität und die Selbstbestimmung schwerstkranker Menschen zu erhalten, zu fördern und zu verbessern und ihnen ein menschenwürdiges Leben bis zum Tod in ihrer vertrauten häuslichen oder familiären Umgebung zu ermöglichen“ [1]. Dieser Anspruch besteht auch für lebenslimitierend erkrankte Kinder und Jugendliche. Ihre besonderen Belange sind der SAPV-Richtlinie des Gemeinsamen Bundesausschusses (G-BA) zufolge zu berücksichtigen. Was diese besonderen Belange beinhalten und was deren Beachtung für die SAPV von Kindern und Jugendlichen (SAPV-KJ) bedeutet, wird in der Richtlinie jedoch nicht weiter erläutert [1].

Aus der Literatur ist bekannt, dass sich die Versorgungsverläufe von Kindern und Jugendlichen in der SAPV von denen erwachsener Palliativpatient*innen unterscheiden: Kinder und Jugendliche werden oftmals über einen längeren Zeitraum versorgt und leiden seltener an onkologischen Erkrankungen [2–4]. Ein Großteil der Kinder und Jugendlichen mit palliativem Versorgungsbedarf hat multiple, komplexe und oftmals seltene Erkrankungen [5]. Viele dieser Kinder und Jugendlichen sind ambulant an sozialpädiatrische Zentren angebunden, in Krisensituationen müssen sie meist ins stationäre Setting wechseln, sofern sie nicht durch ein SAPV-Team versorgt werden [6]. Einige der Kinder und Jugendlichen sind so schwer erkrankt, dass sie ohne SAPV langfristig stationär behandelt werden müssen [7]. Da es in Deutschland nur 2 Kinderpalliativstationen sowie ein Zentrum für altersübergreifende Palliativmedizin gibt, muss ein Großteil der lebenslimitierend erkrankten Kinder und Jugendlichen auf überwiegend kurativ ausgerichteten Stationen versorgt werden [7, 8]. Zudem werden über Kinderhospize neben Trauer- und Sterbebegleitung auch temporäre Entlastungsaufenthalte für Familien lebenslimitierend erkrankter Kinder angeboten [8]. Bisher existieren für Deutschland keine belastbaren epidemiologischen Daten zum palliativen Versorgungsbedarf von Kindern und Jugendlichen. Auf Grundlage von Daten aus dem Vereinigten Königreich wird geschätzt, dass ca. 50.000 Kinder und Jugendliche in Deutschland lebensbedrohlich oder lebenslimitierend erkrankt sind [8, 9]. Basierend auf Abrechnungsdaten wurden im Jahr 2016 in Deutschland 979 Kinder und Jugendliche in der SAPV betreut [10].

In Hessen wird diese Versorgung seit 2013 flächendeckend von 3 SAPV-KJ-Teams erbracht, die auf die Versorgung von Kindern und Jugendlichen im häuslichen Umfeld spezialisiert sind. In einem multiprofessionellen Team behandeln auf pädiatrische Palliativversorgung spezialisierte Pflegekräfte, Ärzt*innen und Fachkräfte anderer Professionen gemeinsam das komplexe Symptomgeschehen mit dem Ziel, die bestmögliche Lebensqualität für Patient*innen zu erreichen. Hierbei werden die Familien unterstützt und angeleitet, die Versorgung unter allen Beteiligten koordiniert und durch die ständige Erreichbarkeit des SAPV-Teams Patient*innen das Leben und Versterben im eigenen Zuhause ermöglicht. Diese Versorgung geschieht derzeit auf Grundlage der SAPV-Richtlinie, die ausgehend von der Versorgungssituation erwachsener Palliativpatient*innen formuliert ist. Die Besonderheiten pädiatrischer Palliativversorgung werden nur sehr vage adressiert, wenn es in § 1 Abs. 4 heißt: „Den besonderen Belangen von Kindern ist Rechnung zu tragen.“

Das Projekt „Evaluation der Spezialisierten Ambulanten Palliativversorgung am Beispiel von Hessen“ (ELSAH; [11]) hat es sich daher unter anderem zum Ziel gesetzt:

1. die in der SAPV-Richtlinie erwähnten „besonderen Belange“ von Kindern und Jugendlichen in der SAPV zu konkretisieren,
2. zu benennen, was diese besonderen Belange für die Erbringung von SAPV-KJ bedeuten, und
3. Empfehlungen zu formulieren, wie diese Belange in der SAPV-Richtlinie stärker berücksichtigt werden können.

Das Projekt wurde vom Innovationsfonds des G‑BA finanziert (FKZ: 01VSF16006) und unter der Konsortialführung des Fachverbands SAPV Hessen am Institut für Allgemeinmedizin Frankfurt und der Abteilung für Allgemeinmedizin, Präventive und Rehabilitative Medizin Marburg, unterstützt durch das Regionalmanagement Nordhessen, von 2017 bis 2020 durchgeführt. Nähere Informationen zu den Arbeitspaketen des Projekts sind den Studienprotokollen zu entnehmen [11, 12].

In dieser Publikation stellen wir die Gesamtergebnisse der ELSAH-Studie zu den besonderen Belangen von Kindern und Jugendlichen in der SAPV vor. Wir fassen dabei die Ergebnisse der unterschiedlichen Erhebungsphasen (Fragebogenerhebungen, qualitative Interviews, teilnehmende Beobachtungen und Fokusgruppendiskussionen mit Leistungserbringer*innen, Angehörigen und Patient*innen der Erwachsenen-SAPV und SAPV-KJ in Hessen sowie der Auswertung von Dokumentationsdaten aller hessischen SAPV-Teams) zusammen und stellen die besonderen Belange von Kindern, Jugendlichen und ihren Familien in der SAPV-KJ dar. Auf Basis der Studienergebnisse formulieren wir Empfehlungen zur Überarbeitung der SAPV-Richtlinie, damit diese künftig die Belange von Kindern, Jugendlichen und deren Familien adäquat adressiert.

## Methoden

Um die besonderen Belange von Kindern und Jugendlichen in der SAPV zu analysieren, wurden in der ELSAH-Studie in einem sequenziellen Mixed-Methods-Design verschiedene Datenerhebungen durchgeführt, die einen multiperspektivischen Blick auf die besonderen Belange von Kindern, Jugendlichen und ihren Familien in der SAPV ermöglichten [11].

**Figure 1:**
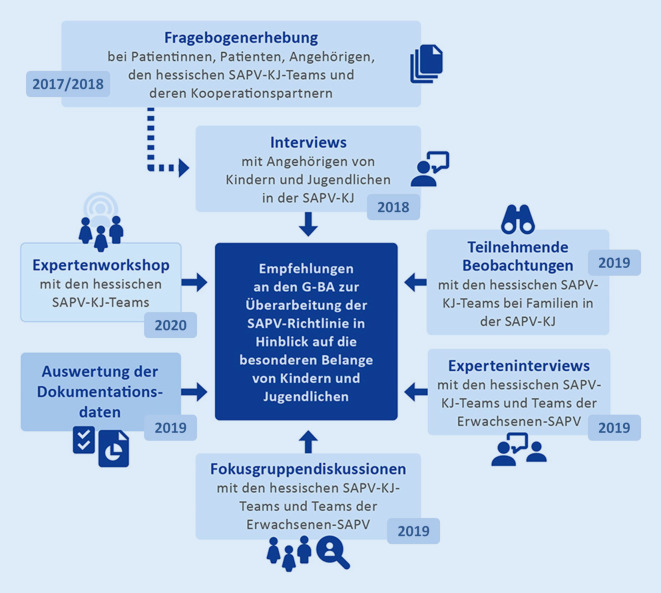

Auf Grundlage der zusammengeführten Ergebnisse (Übersicht der Datenerhebungen Abb. 1) wurden Empfehlungen zur Überarbeitung der SAPV-Richtlinie erarbeitet.

### Rekrutierung

Die Rekrutierung der Studienteilnehmenden aus Erwachsenen-SAPV^1^ und SAPV-KJ-Teams erfolgte in Kooperation mit der Abteilung für Allgemeinmedizin der Philips-Universität Marburg und dem Fachverband SAPV Hessen. Alle hessischen Teams der Erwachsenen-SAPV (*n* = 22 Teams) und der SAPV-KJ (*n* = 3 Teams) nahmen an der Studie teil.

Patient*innen und deren Familien wurden mit Unterstützung der SAPV-KJ-Teams rekrutiert. Alle Teilnehmenden (bzw. deren Sorgeberechtigte) wurden sowohl schriftlich als auch mündlich über die Studie informiert und willigten schriftlich in die Studienteilnahme ein. Informationen zu den Teilnehmenden der verschiedenen Datenerhebungen sind Tab. 1 zu entnehmen.

**Table 1:** 

Datenerhebung	Teilnehmer*innen	Auswertungsstrategie
Fragebogen zur Einschätzung der derzeitigen Versorgungssituation in der SAPV-KJ (sprachlich an die jeweilige Teilnehmer*innengruppe angepasst)	41 Leistungserbringer*innen (SAPV-KJ und Kooperationspartner*innen) 28 Angehörige über 18 Jahre 6 Angehörige unter 18 Jahre 2 Patient*innen	*Deskriptive statistische Auswertung* Die Daten wurden mittels des Statistikprogramms SPSS 25 deskriptiv hinsichtlich der Häufigkeiten der jeweiligen Antwortkategorien ausgewertet
Narrative Interviews [13]	9 Interviews mit 13 Angehörigen lebenslimitierend erkrankter Kinder, die in der SAPV-KJ versorgt werden oder wurden (9 Mütter, 4 Väter)	*Auswertung nach Grounded Theory *[7, 14] Alle Interviews wurden in einem Team von 3 in qualitativen Methoden ausgebildeten Forscher*innen fallspezifisch und fallübergreifend analysiert. Jeder Fall wurde mit dem Ziel des ständigen Vergleichs in 6 Schritten analysiert 1. Offenes Codieren: Das Interview wurde induktiv im Detail codiert, unterstützt durch die Software MAXQDA [15] 2. Axiales Codieren: Die entwickelten Codes wurden zu breiteren Kategorien und Konzepten zusammengefasst und diese wurden mit Kategorien und Konzepten aus früheren Fällen verglichen und zusammengeführt 3. Memos: Überlegungen, Zusammenhänge und Fragen, die sich aus dem offenen und axialen Codieren ergaben, wurden in Memos festgehalten 4. Codierparadigma: Es wurde eine Mindmap erstellt, die auf dem Codierparadigma der Grounded Theory basiert 5. Fallbericht: Ein Fallbericht, der die Schritte 1–4 sowie grundlegende Informationen über die Familiensituation und den Gesundheitszustand des Kindes oder Jugendlichen zusammenfasst wurde verfasst 6. Mindmap: Auf der Basis der vorangegangenen Schritte wurde eine Mindmap erstellt, die die Hauptkonzepte und Kategorien des Falls, die Zusammenhänge zwischen diesen Konzepten und Verbindungen zu anderen Fällen darstellt Nachdem die Schritte 1 bis 6 für jeden Fall durchgeführt wurden, wurde eine umfassende Ergebnismap erstellt, die alle fallübergreifend relevanten Konzepte umfasst
Teilnehmende Beobachtungen	Begleitung der 3 hessischen SAPV-KJ-Teams bei 8 Hausbesuchen (bei 8 verschiedenen Familien)	*Thematische Analyse *[16] Während der teilnehmenden Beobachtungen wurden Feldnotizen angefertigt, die später zu Beobachtungsprotokollen ausgearbeitet wurden. 2 Forscherinnen analysierten die Beobachtungsprotokolle induktiv im Hinblick auf die Versorgungspraktiken der SAPV-KJ-Teammitglieder in der Zusammenarbeit mit den Eltern. Die Forscherinnen codierten das Material mit Unterstützung der Software MAXQDA [15], diskutierten ihre Codes und entwickelten ein endgültiges Codesystem, das für alle Beobachtungsprotokolle verwendet wurde. Während der Analyse wurden Kategorien erarbeitet, die die Versorgungspraktiken und Verhaltensweisen der SAPV-KJ-Teams abbilden
Leitfadengestützte Telefoninterviews	28 Interviews (16 Pflegekräfte, 12 Ärzt*innen) mit 14 Personen aus SAPV-KJ-Teams und 14 Personen aus Teams der Erwachsenen-SAPV	*Thematische Analyse *[16] Es erfolgte eine Auswertung der Interviews durch 3 Projektmitarbeiterinnen. Zunächst wurden deduktiv Oberkategorien aus den Themen des Leitfadens abgeleitet, die dann im Laufe der Auswertung durch induktive Kategorien ergänzt wurden. In einem iterativen Prozess wurden die Codes und Codierungen verglichen, diskutiert, zu einem gemeinsamen Codesystem zusammengeführt und auf das gesamte Material angewendet. In der finalen Auswertung wurden die Interviews schließlich nach SAPV-KJ und SAPV für Erwachsene gruppiert und kontrastiert. 2 Forscherinnen arbeiteten dabei heraus, welche Unterschiede sich in den Ausprägungen der einzelnen Kategorien in den beiden Gruppen zeigen
Fokusgruppendiskussionen	3 Fokusgruppendiskussionen (11 Pflegekräfte, 8 Ärzt*innen) mit insgesamt 19 Personen aus Erwachsenen-SAPV und SAPV-KJ	*Thematische Analyse *[16] Die Auswertung erfolgte durch 3 Projektmitarbeiterinnen. Ausgehend von der Forschungsfrage wurde das Datenmaterial in einem induktiven Prozess mit Unterstützung der Software MAXQDA [15] codiert. Nach Entwicklung von initialen Codes wurden die zugrunde liegenden Themen analysiert und in übergeordneten Kategorien zusammengefasst. Die gebildeten Kategorien wurden im Sinne eines zirkulären Forschungsprozesses auf das gesamte Material angewendet
Expertenworkshop	28 Leistungserbringer*innen (21 Leistungserbringer*innen aus Teams der Erwachsenen-SAPV, 7 aus Teams der SAPV-KJ)	*Kommunikative Validierung der Ergebnisse *[17] Im Rahmen eines Expert*innenworkshops wurden zunächst ärztlichen, pflegerischen und psychosozialen Leistungserbringer*innen der hessischen SAPV-KJ die im Projekt erarbeiteten Empfehlungen als Entwurf vorgelegt. Die Leistungserbringer*innen diskutierten die Empfehlungen und gaben Anregungen, insbesondere im Hinblick auf Formulierungen. In einem zweiten Schritt wurden die Empfehlungen mit Leistungserbringer*innen der SAPV-KJ und der Erwachsenen-SAPV gemeinsam diskutiert. Anmerkungen und Änderungsvorschläge zu Formulierungen wurden im Nachgang von den Forscherinnen unter Betrachtung der Projektergebnisse geprüft und in die Empfehlungen eingearbeitet
Auswertung der Dokumentationsdaten	Der Auswertung liegen die anonymisierten Dokumentationsdaten aller hessischen SAPV und SAPV-KJ-Teams zugrunde, die von 2014/1. Halbjahr bis 2018/2. Halbjahr an den Fachverband SAPV Hessen e. V. übertragen wurden	*Deskriptive Auswertung der SAPV-begründenden Diagnosen* In der Kategorie „Kinder und Jugendliche“ finden sich alle Versorgungsfälle von Patient*innen ≤ 18 Jahre sowie alle diejenigen über 18 Jahre, die durch ein SAPV-KJ-Team versorgt wurden (*n* = 830). In der Kategorie „Erwachsene“ finden sich alle Versorgungsfälle von Patient*innen über 18 Jahre, die von einem Team der Erwachsenen-SAPV versorgt wurden (*n* = 70.601). Die Versorgungsfälle entsprechen nicht der Anzahl von Patient*innen. Da bei jeder SAPV-Neuverordnung z. B. nach einem Krankenhausaufenthalt ein neuer Versorgungsfall generiert wird, können Patient*innen mehrere Versorgungsfälle auslösen. Sämtliche ICD-10-Codes der SAPV-begründenden Diagnosen wurden einer ICD-10-Überkategorie z. B. C00-D48 (Neubildungen) zugeordnet. Die 5 häufigsten SAPV-begründenden Diagnosen in der Gruppe der Kinder und Jugendlichen werden als prozentualer Anteil an allen SAPV-auslösenden Diagnosen in dieser Gruppe dargestellt und dem prozentualen Anteil in der Gruppe der Erwachsenen gegenübergestellt

*ICD-10* International Statistical Classification of Diseases and Related Health Problems (Version 10), *SAPV* Spezialisierte Ambulante Palliativversorgung, *SAPV-KJ* Spezialisierte Ambulante Palliativversorgung für Kinder und Jugendliche

### Auswertung und Synthese der Ergebnisse

Die Auswertungsstrategien der einzelnen Erhebungen sind in Tab. 1 dargestellt. Detaillierte Ausführungen zu Methodik und Ergebnissen der einzelnen Projektschritte wurden an anderer Stelle veröffentlicht [7, 11, 18, 19]. In systematischen Diskussionen wurden die Ergebnisse der verschiedenen Datenerhebungen im Forscherinnenteam synthetisiert, um auf dieser Grundlage Empfehlungen für die Überarbeitung der SAPV-Richtlinie zu formulieren. Sowohl die Ergebnisse als auch die Empfehlungen wurden im gesamten Projektkonsortium diskutiert und abschließend von Leistungserbringer*innen der SAPV-KJ kritisch kommentiert sowie in einem letzten Schritt von Leistungserbringer*innen der SAPV-KJ und der Erwachsenen-SAPV gemeinsam diskutiert. Die Ergebnisse wurden auf diese Weise kommunikativ validiert und die Leistungserbringer*innen gaben Anregungen, insbesondere im Hinblick auf Formulierungen in den Empfehlungen. Diese wurden im Nachgang von den Forscherinnen unter Betrachtung der Projektergebnisse geprüft, eingearbeitet und im gesamten Projektkonsortium abschließend diskutiert.

## Ergebnisse

Im Folgenden sind die zusammengeführten Ergebnisse aller Datenerhebungen aus dem Arbeitspaket 2 des ELSAH-Projekts dargestellt. Detailergebnisse zu den einzelnen Erhebungen wurden an anderer Stelle ausführlich beschrieben [7, 18, 19]. Zunächst werden die besonderen Belange von Kindern, Jugendlichen und ihren Familien in der SAPV dargestellt sowie die besonderen Anforderungen, die diese Belange an die Erbringung der SAPV-KJ stellen. Danach fassen wir zusammen, wie beides in die SAPV-Richtlinie integriert werden sollte.

### Palliative Versorgung bei Erkrankungen des Kindes- und Jugendalters

**Figure 2:**
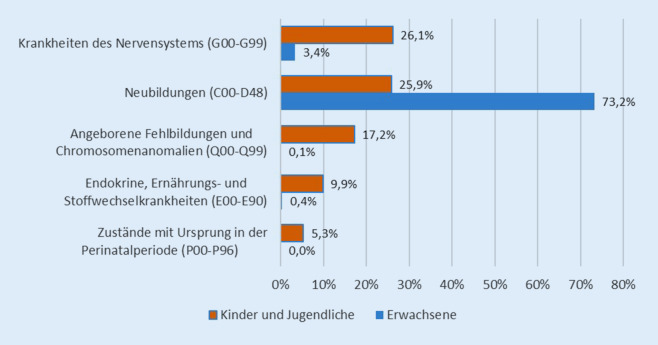

Die Dokumentationsdaten der hessischen SAPV- und SAPV-KJ-Teams zeigen, dass die SAPV-begründenden Diagnosen bei Kindern und Jugendlichen vielfältiger sind als bei Patient*innen in der Erwachsenen-SAPV, die zu 73 % aufgrund einer Krebserkrankung palliativ versorgt werden (Abb. 2).

Besonders in den narrativen Interviews mit Angehörigen wurde deutlich, dass die palliative Versorgung von Patient*innen mit komplexen oftmals seltenen Erkrankungen des Kindes- und Jugendalters eine besondere Expertise erfordert. 4 von 9 Patient*innen aus den Angehörigeninterviews (AI) litten an einer seltenen Erkrankung, 2 hatten keine abschließende Diagnose.

Da die Verstoffwechselung von Medikamenten häufig anders erfolgt als bei Erwachsenen, insbesondere bei Vorliegen einer Stoffwechselerkrankung, benötigen die Familien Expert*innen für Schmerzmedikation und weitere Therapiemaßnahmen bei Kindern und Jugendlichen, um eine gelungene Kontrolle des komplexen Symptomgeschehens zu erreichen:

Die meisten Ärzte kennen die Erkrankung ja gar nicht, weil sie einfach so selten ist. Mein Sohn verstoffwechselt im Prinzip jedes Medikament so, wie er es eigentlich gerade nicht sollte. Und diese ganze spezielle Thematik kennt eigentlich nur das SAPV-Team (Mutter eines SAPV-KJ-Patienten, AI).

In den Fokusgruppendiskussionen (FG) machten auch Leistungserbringer*innen aus der Erwachsenen-SAPV deutlich, dass sie für die SAPV von Patient*innen mit den oben beschriebenen Krankheiten des Kindes- und Jugendalters nicht ausgebildet sind:

Als Erwachsenenmedizinerin würde ich mir nie anmaßen, irgendwelche komplexen Syndrome des Kindesalters zu behandeln. Never ever (Ärztin aus einem SAPV-Team, FG).

In den teilnehmenden Beobachtungen wurde darüber hinaus die besondere Herausforderung in der Kommunikation mit Kindern deutlich, insbesondere mit Patient*innen, die sich selbst verbal nicht äußern können. Fähigkeiten hinsichtlich kindgerechter Sprache sowie des „Lesens“ von Mimik und Körpersprache waren hierbei unabdingbar.

### Familienzentrierte Versorgung

In den Experteninterviews (EI) und Fokusgruppendiskussionen (FG) mit den Leistungserbringer*innen wurde deutlich, dass Angehörige sowohl in der SAPV als auch der SAPV-KJ eine wichtige Rolle in der Versorgung der Patient*innen und als Ansprechpartner*innen für die Teams spielen. In der SAPV-KJ kommt der familienzentrierten Versorgung allerdings eine besondere Bedeutung zu, da die Stellvertreterrolle der Eltern besonders ausgeprägt ist. Die Patient*innen in der SAPV-KJ sind überwiegend minderjährig, meist schwerstmehrfachbehindert und können oft nicht selbst für sich sprechen:

Die Schwierigkeit ist, diese Patientenklientel kann man nicht fragen. Das sind häufig neurologisch Erkrankte, also sind die Ansprechpartner die Eltern (Pflegefachkraft aus einem SAPV-KJ-Team, FG).

Sowohl in der SAPV-KJ als auch in der SAPV spielt die Stabilisierung des Familiensystems eine zentrale Rolle, um die Lebensqualität der Patient*innen zu erhöhen bzw. zu erhalten und eine effektive Behandlung zu ermöglichen. Insbesondere Leistungserbringer*innen der SAPV-KJ betonten, wie wichtig es sei, ein Vertrauensverhältnis zu den Eltern aufzubauen. Dies sei zeitintensiv, aber absolut notwendig, um bei der Versorgung des Kindes gut zusammenzuarbeiten:

Die SAPV muss auf eine Art und Weise passieren, die wertschätzend ist und die unterstützend ist für die Familie. Die Familie muss befähigt werden, zu Koproduzenten dieser guten Versorgung zu werden (Arzt aus einem SAPV-KJ-Team, FG).

Sowohl die Angehörigen- als auch die Experteninterviews zeigten, dass die Befähigung der Angehörigen durch die SAPV-KJ-Teams aufgrund der unklaren Krankheitsverläufe und Prognosen sowie der wechselnden Gesundheitszustände der erkrankten Kinder und Jugendlichen durch Anleitung und Gespräche immer wieder neu hergestellt werden muss. Behandlungsziele und Erwartungen müssen fortwährend überdacht und angepasst werden, was Zeit und Einfühlungsvermögen voraussetzt. Die Eltern werden von den Teams außerdem zur Versorgung ihres Kindes (z. B. der Medikamentengabe, dem Absaugen von Atemwegssekret, alternativen Methoden der Symptomlinderung) angeleitet. Die Angehörigeninterviews zeigten, dass diese Anleitung und Befähigung zur Versorgung der Kinder für die Eltern einen sehr großen Stellenwert hat und ihnen bei der Bewältigung der Situation hilft. Sie fühlen sich hierdurch selbstwirksam und weniger hilflos und ausgeliefert:

Ja und das hat auch einfach geholfen, dass man sich für sein eignes Kind wieder zuständig fühlen kann (Mutter einer SAPV-KJ-Patientin, AI).

Insbesondere für Familien mit Geschwisterkindern war die SAPV eine große Entlastung, um die Versorgung ihres kranken Kindes und das Leben als Familie zu vereinbaren:

Wenn ich mich hätte entscheiden müssen am Abend, wenn ich von der Arbeit nach Hause komme, welches Kind ich sehe: Fahr ich noch mal ins Krankenhaus oder fahr ich nach Hause? Das wäre nix gewesen, das hätte ich mir nicht vorstellen können (Vater einer SAPV-KJ-Patientin, AI).

### Besonders aufwendige psychosoziale Versorgung

Sowohl die Teams der Erwachsenen-SAPV als auch der SAPV-KJ behandeln ihre Patient*innen nicht nur medizinisch-pflegerisch, sondern versorgen sie und ihre Angehörigen auch psychosozial. In den Einzelinterviews und in den Fokusgruppendiskussionen erklärten die Leistungserbringer*innen, dass sie sich hierfür die Unterstützung durch einen Psychologen oder eine Psychologin im Team wünschen. Leistungserbringer*innen beider SAPV-Bereiche waren sich einig, dass das Versterben eines Kindes eine wesentlich größere Ausnahmesituation darstellt als das Versterben eines (älteren) Erwachsenen und mit einer ganz besonderen Belastung der Angehörigen (und der Versorger*innen) einhergeht.

Ein Sterben von Kindern ist immer problematisch, egal welche Krankheit das ist. Da ist immer eine extreme Symptomlast, und wenn sie rein sozial ist (Arzt aus einem SAPV-KJ-Team, FG).

Die Angehörigeninterviews und die teilnehmenden Beobachtungen machten deutlich, dass die Eltern das SAPV-KJ-Team auch als Ansprechpartner für ihre Sorgen und psychischen Belange betrachten.

Als es meinem Sohn so schlecht ging, sind mir dann auch einige Tränen geflossen. Da wird man in den Arm genommen. Die haben sich nicht nur um das Kind gekümmert, auch um die psychisch abgeschlagenen Eltern (Mutter eines SAPV-Patienten, AI).

Auch nach dem Versterben des Kindes begleiten die SAPV-KJ-Teams die Eltern häufig weiter, indem sie Nachgespräche führen und an Beerdigungen und Trauerfeiern teilnehmen. Die Eltern äußerten in den Interviews ebenfalls, dass es ihnen wichtig ist, sich mit den Teammitgliedern, die Krankheit und Versterben des Kindes miterlebt haben, auszutauschen. Insbesondere in den Experteninterviews berichteten Leistungserbringer*innen, dass es nicht dem tatsächlichen Bedarf und Versorgungsgeschehen entspricht, dass die Leistungsvergütung für die SAPV sozialrechtlich mit dem Tod der Patient*innen endet. Alle Leistungen nach dem Versterben des Kindes müssen ehrenamtlich erbracht werden:

Von der Krankenkasse aus gesehen endet SAPV mit dem letzten Atemzug des Kindes und dann ist Schluss. Und selbst die Leichenschau ist eine Privatleistung. Also so ist das Gesetz, aber so kann man ja nicht arbeiten. Also zu über 90 % möchten die Eltern ein Nachgespräch, die möchten den Kontakt halten. Es kommen viele zu uns am Tag der Offenen Tür zum Beispiel. Aber das ist auf reiner Spendenbasis, aber das ist unabdingbar, ja? Sowohl für die Familien, als auch für unser Team (Pflegefachkraft aus einem SAPV-KJ-Team, EI).

### Besonders aufwendige Koordination der beteiligten Versorger*innen

Die Angehörigeninterviews sowie die Einzelinterviews und Fokusgruppen mit den Leistungserbringer*innen zeigten, dass die Koordination der beteiligten Versorger*innen bei Kindern und Jugendlichen neben Krankenkassen, Fachspezialist*innen, Apotheken, Logo- oder Ergotherapie und vielen mehr auch den Einbezug von zusätzlichen nichtmedizinischen Institutionen umfassen kann, wie etwa die Schule oder den Kindergarten. Zudem sind Spezialist*innen für seltene Erkrankungen häufig räumlich weit voneinander entfernt. Um die Kontinuität der Versorgung zu gewährleisten, ist die Abstimmung zwischen den Versorger*innen jedoch besonders wichtig:

Diese Patienten sitzen in einem Behandlungsgeflecht. Da ist das SPZ (Sozialpädiatrisches Zentrum), also ein externer Experte, der mit denen via E‑Mail konferiert. Und wir erleben auch, dass Kinder im Krankheitsverlauf in eine Spezialklinik gehen. Das macht es für uns immer wieder aufwendig, runde Tische einzuberufen (Arzt aus einem SAPV-KJ-Team, EI).

Den Mitgliedern der SAPV-KJ-Teams kommt dabei in der Regel eine koordinierende Rolle zu:

Die Arbeit mit den Rezepten, sich drum kümmern, dass das wirklich funktioniert mit der Krankengymnastik, dass die heimgekommen ist … Oder wenn die Krankenkasse ewig braucht um irgendwelche Therapiestunden zu genehmigen, dann rufen die da an. Also vieles könnte ich hier am Tag so alleine gar nicht leisten (Eltern eines SAPV-Patienten, AI).

### Angeleiteter Erstkontakt zur SAPV-KJ

In den Angehörigeninterviews berichteten viele Familien, dass sie nach Diagnosestellung ohne Information über die Möglichkeit der SAPV-KJ aus dem Krankenhaus entlassen wurden:

Wir kriegten bei Entlassung aus dem Krankenhaus nichts in die Hand, nicht von der Lebenshilfe, nicht vom Pflegedienst, gar nichts. Wir wurden da im Grunde auf die Straße gesetzt und hatten eine Tube Diazepam dabei (Mutter eines SAPV-KJ-Patienten, AI).

Wir kannten das vorher gar nicht, dass es sowas wie die SAPV-KJ gibt. Die Ärzte im Krankenhaus haben uns davon nichts erzählt (Mutter einer SAPV-KJ-Patientin, AI).

Die Eltern mussten ihr Kind zunächst ohne Hilfe zu Hause versorgen, was z. T. zu einer extremen Belastungssituation führte. Ein Großteil der interviewten Eltern berichtete, dass sie „zufällig“, z. B. über Bekannte, einen Pflegedienst oder Selbstrecherche im Internet von der SAPV-KJ erfahren haben. Auch in den Experteninterviews mit den Leistungserbringer*innen wurde deutlich, dass ein großes Informationsdefizit bezüglich der Existenz der SAPV-KJ und ihrer Arbeit besteht. Ebenso berichteten Leistungserbringer*innen, dass eine erste Kontaktaufnahme bereits vor der Geburt des Kindes sinnvoll sei, um die Eltern vorzubereiten und zu unterstützen:

Also die Menschen müssen wissen, dass es eine Struktur um die Geburt gibt, die diesen palliativen Weg mitgehen kann. Ja? Und dass man nicht zwingenderweise auf der Neo-Intensiv endet (Arzt aus einem SAPV-KJ-Team, FG).

Zahlreiche Leistungserbringer*innen berichteten, dass sie selbstständig andere Versorger*innen ansprechen und durch Vorträge, Fortbildungen, Social-Media-Aktivitäten und Pressearbeit versuchen, das Wissen über die SAPV-KJ in der Öffentlichkeit zu verbessern.

### Versorgungslücke zwischen Grund‑/Regelversorgung und SAPV für Kinder und Jugendliche

Die Datenerhebungen mit Eltern und Leistungserbringer*innen zeigen deutlich, dass die Teilnehmenden eine Versorgungslücke für schwerstkranke Kinder und Jugendliche zwischen Grund‑/Regelversorgung und der SAPV-KJ erleben. Die Angehörigen von Kindern und Jugendlichen berichteten häufig, dass sie die SAPV-KJ als einzige qualifizierte ärztliche Versorgungsmöglichkeit betrachten. Bevor sie eine Versorgung durch ein SAPV-KJ-Team erhielten, mussten sie oft und teilweise auch wegen kleinerer Belange, wie z. B. dem Absaugen der Lunge, ins Krankenhaus fahren, wo sie dann stationär aufgenommen wurden:

Wir waren vorher beim normalen Kinderarzt, und da haben wir immer eine Einweisung bekommen, egal was unser Kind hatte, wir hatten eine Einweisung. Und so waren wir alle vier Wochen in der Klinik. Ein bisschen Schnupfen, zack, eine Einweisung. Es könnte ja eine Lungenentzündung werden (Mutter eines SAPV-Patienten, AI).

Die Fahrt in ein Krankenhaus ist für die Kinder, die größtenteils nicht mobil sind und gerätemedizinisch versorgt werden, mit großem Aufwand verbunden. Der Aufenthalt im Krankenhaus bedeutet für die Patient*innen und ihre Familien Stress, unerwünschte diagnostische Maßnahmen und die Gefahr nosokomialer Infektionen. Nichtsdestotrotz war – vor der Betreuung durch ein SAPV-KJ-Team – eine Klinik für die interviewten Familien oftmals die einzige Versorgungsmöglichkeit. Es fehlt häufig der Zugang zu einer (aufsuchenden) ambulanten palliativen Regelversorgung für Kinder. Auch in den Interviews mit Leistungserbringer*innen wurde diese Versorgungslücke klar benannt:

Es gibt eine Versorgungslücke für diese schwerstkranken, chronisch kranken Patienten, für die es manchmal keine andere Chance gibt, als in die Klinik zu gehen bei einem banalen Infekt, weil es einfach kein aufsuchendes Unterstützungssystem für zuhause gibt (Ärztin aus einem SAPV-KJ-Team, EI).

Insbesondere für Kinder und Jugendliche mit komplexen Erkrankungen, die die Anforderungen für eine SAPV aber (noch) nicht erfüllen, fehlt eine allgemeine (aufsuchende) ambulante Versorgungsstruktur, auf die in unklaren Situationen oder Krisen zurückgegriffen werden kann.

### Empfehlungen zur Überarbeitung der SAPV-Richtlinie

Aus den aufgeführten besonderen Belangen und Anforderungen von Kindern, Jugendlichen und ihren Familien können Empfehlungen abgeleitet werden, die in der Infobox 1 zusammenfassend dargestellt sind.

## Diskussion

„Kinder sind keine kleinen Erwachsenen“ – dieser Leitspruch der Pädiatrie trifft umso mehr auf die SAPV von Kindern und Jugendlichen zu. Sie leiden an komplexen, oftmals seltenen Erkrankungen und bedürfen einer besonders aufwendigen Palliativversorgung durch ein Team mit pädiatrischer Expertise. Die SAPV-KJ muss die gesamte Familie einbeziehen und die oftmals überregional verteilte Versorgung koordinieren. Die große Unsicherheit aufgrund unklarer Prognosen und Krankheitsverläufe sowie die Ausnahmesituation, dass ein Kind vor den Eltern versterben wird und Angehörige oft auch minderjährige Geschwisterkinder sind, macht eine besonders aufwendige psychosoziale Versorgung von Patient*innen und Angehörigen notwendig. Zudem wurde im Rahmen der ELSAH-Studie deutlich, dass die Möglichkeit, Unterstützung durch SAPV-Teams für Kinder und Jugendliche zu erhalten, wenig bekannt ist und die Wege für die Familien in die SAPV-KJ oftmals nicht gebahnt werden. Für lebenslimitierend erkrankte Kinder und Jugendliche, die zwar einer aufsuchenden Palliativversorgung bedürfen, jedoch keinen Bedarf an einer so intensiven Betreuung wie in der SAPV haben, besteht zudem eine Versorgungslücke.

Die Ergebnisse und Empfehlungen der ELSAH-Studie stehen in Einklang mit den IMPaCCT-Standards pädiatrischer Palliativversorgung in Europa [20]. Durch unsere Studie wurden die besonderen Belange von Kindern und Jugendlichen für die Versorgungsform SAPV herausgestellt. Eine rechtsverbindliche Festschreibung dieser Belange und der damit verbundenen notwendigen Versorgungsleistungen gewährleistet die adäquate Versorgung von Kindern und Jugendlichen in der SAPV sowie die Vergütung dieser Leistungen. Das SAPV-KJ-Team bietet für viele Familien von lebenslimitierend erkrankten Kindern und Jugendlichen die Unterstützung, die ein gemeinsames bestmögliches Familienleben mit der Erkrankung und dem bevorstehenden Versterben des Kindes (wieder-)ermöglicht [7]. Umso wichtiger ist es, professionelle Versorger*innen bezüglich der Möglichkeiten und Kompetenzen der SAPV-KJ zu sensibilisieren, damit Familien frühzeitig von dieser Versorgungsform erfahren und die notwendige Unterstützung erhalten können. Anders als in der Erwachsenenversorgung, wo eine Überleitung von den onkologischen Stationen oder ambulanten Versorger*innen gebahnt wird, ist der Zugang zur SAPV-KJ für die berechtigte Patientengruppe und ihre Angehörigen oftmals schwieriger oder wird teilweise nicht ermöglicht.

Die frühe Begleitung der Eltern nach einer vorgeburtlich diagnostizierten lebenslimitierenden Erkrankung ihres Kindes sowie die Trauerbegleitung der Eltern durch das SAPV-Team stoßen auf sozialrechtlich bedingte Hürden, die eine Vergütung dieser Leistungen erschweren. In diesen Fällen ist der/die SAPV-berechtigte Patient*in entweder noch nicht geboren oder bereits verstorben und die Eltern haben selbst keinen Anspruch auf SAPV-Leistungen. Auch der Einsatz eines ambulanten Kinderpalliativteams im stationären Setting ist durch die strikte Trennung der Sektoren erschwert. Eine erste Kontaktaufnahme durch das spezialisierte ambulante Palliativteam im stationären Setting ist in vielen Fällen jedoch unabdingbar, um den Übergang in die Häuslichkeit zu planen. Das Wissen der Teams zum individuellen Einzelfall und das im Versorgungszeitraum gewachsene Vertrauensverhältnis stellen eine wichtige Ressource in der Trauerbegleitung der Eltern dar, die jedoch ebenfalls nicht vergütet wird. Hier sollte die Möglichkeit eines Kostenausgleichs zwischen dem Kostenträger der Kinder und Jugendlichen und dem Kostenträger der Angehörigen geschaffen werden.

In der SAPV für Erwachsene spielt die Abgrenzung zur allgemeinen ambulanten Palliativversorgung (AAPV) eine große Rolle [21]. Die AAPV wird in der Regel von Hausärzt*innen gegebenenfalls in Kooperation mit geschulten Pflegediensten erbracht. Sie richtet sich an schwerstkranke und sterbende Patient*innen, die jedoch nicht an einem komplexen Symptomgeschehen leiden und deren Versorgung deshalb im häuslichen Setting durch Hausärzt*innen und Pflegedienst erbracht werden kann. Wie unsere Studienergebnisse zeigen, besteht für Kinder und Jugendliche keine vergleichbare Versorgungsstruktur. Aufgrund dieser Versorgungslücke müssen sich schwerst- und komplex erkrankte Kinder und Jugendliche, die nicht in der SAPV versorgt werden, oftmals stationär versorgen lassen, auch wenn das Gesundheitsproblem eine solche Versorgung eigentlich nicht begründet [7]. Um die Krankenhauseinweisungen von schwerstkranken Kindern und Jugendlichen zu verringern, Familien die damit verbundenen Belastungen zu ersparen und die Kontinuität der Versorgung zu gewährleisten, ist es unbedingt notwendig, die Anspruchsvoraussetzungen der SAPV-KJ auszudehnen oder eine weitere, niedrigschwellige Versorgungsform zu etablieren. Eine solche Versorgungsform muss Hausbesuche durch ein Team mit pädiatrischer Expertise bei akuten Gesundheitsproblemen beinhalten.

Sämtliche Empfehlungen, die im ELSAH-Projekt für die Anpassung der SAPV-Richtlinie in Hinblick auf die besonderen Belange von Kindern und Jugendlichen erarbeitet wurden, gelten auch für einen möglichen SAPV-Bundesrahmenvertrag für Kinder und Jugendliche.

### Stärken und Limitationen

Die ELSAH-Studie wurde in der Ethikkommission des Universitätsklinikums Frankfurt beraten und ein positives Ethikvotum erteilt. Uns war die besondere Situation der betroffenen Familien bewusst. Wir haben deshalb den Rekrutierungsweg über die SAPV-KJ-Teams gewählt, die die gesundheitliche Lage und Belastungssituation in den Familien am besten einschätzen konnten. Gleichzeitig kann dieser Weg zu einem Selektionsbias geführt haben. In unserer Stichprobe sind kaum Patient*innen der SAPV-KJ selbst zu Wort gekommen, was vor allem daran liegt, dass die Kinder entweder noch sehr jung waren oder sich aufgrund der meist neuropädiatrischen Erkrankungen selbst nicht äußern konnten. Für ein Kind oder einen Jugendlichen kann das Sprechen über das eigene Versterben zudem eine besondere Belastung darstellen, weshalb Studienteam, SAPV-KJ-Teams, Angehörige und Patient*innen diesbezüglich sehr zurückhaltend waren. Die Interaktion zwischen SAPV-KJ-Teams, Eltern und Patient*innen konnten wir jedoch im Rahmen der teilnehmenden Beobachtungen erfassen.

Stärken der ELSAH-Studie sind die Verknüpfung unterschiedlicher Erhebungs- und Analysemethoden sowie die Integration der Perspektiven von Angehörigen, Leistungserbringer*innen der Erwachsenen-SAPV und der SAPV-KJ. In diesem Artikel wurden die Ergebnisse der unterschiedlichen Erhebungsphasen miteinander verbunden, um auf diese Weise einen Gesamteindruck zu ermöglichen. Im gesamten Projekt waren uns zudem partizipative Ansätze wichtig. So wurden die hier präsentierten Ergebnisse Leistungserbringer*innen aus der Erwachsenen-SAPV und der SAPV-KJ vorgestellt und mit ihnen diskutiert. Neben wissenschaftlichen Publikationen [7, 18] wurden die Studienergebnisse zudem in einer laienverständlichen Ergebnisbroschüre [22] und einem Abschlussfilm [23] aufbereitet und den Studienteilnehmer*innen und der (Fach‑)Öffentlichkeit zugänglich gemacht.

## Fazit

Die spezialisierte ambulante Palliativversorgung von Kindern und Jugendlichen sowie von volljährigen Patient*innen, die seit dem Kindes- und Jugendalter erkrankt sind, bedarf einer eigenständigen Versorgungsform mit Vergütungsmodalitäten, die den besonderen Versorgungsaufwand abbilden.

### Infobox 1: Empfehlungen zur Anpassung der SAPV-Richtlinie für Kinder und Jugendliche

- Die SAPV-KJ muss in einer eigenständigen Versorgungsstruktur von SAPV-KJ-Teams erbracht werden, deren Teammitglieder neben palliativmedizinischer bzw. -pflegerischer auch über pädiatrische Expertise verfügen.
- Als Ziel und Inhalt der SAPV-KJ sollten festgeschrieben werden:
  - eine familienzentrierte Versorgung,
  - die besonders aufwendige Beratung, Anleitung und Begleitung der Sorgeberechtigten,
  - die besonders aufwendige psychosoziale Versorgung,
  - die besonders aufwendige Koordination der spezialisierten palliativmedizinischen und palliativpflegerischen Versorgung.
- Für einen angemessenen Übergangszeitraum und mit dem Ziel einer Überleitung in bestehende Strukturen sollte die Trauerbegleitung von Angehörigen durch das betreuende SAPV-KJ-Team geleistet werden.
- Familien, bei deren Kind (vorgeburtlich) eine lebenslimitierende Erkrankung im Krankenhaus diagnostiziert wird, sollten (nachgeburtlich) eine strukturierte Überleitung ins häusliche Setting erhalten.
- Die Versorgungslücke für schwerstkranke Kinder und Jugendliche sollte durch eine ambulante, aufsuchende Palliativversorgung geschlossen werden.
- Die hier formulierten besonderen Belange treffen auf schwerstkranke und sterbende Kinder und Jugendliche sowie volljährige Patient*innen, die seit dem Kindes- und Jugendalter erkrankt sind, zu.

## Footnotes

1 Die Erbringung von SAPV ist für erwachsene Patient*innen sowie Kinder und Jugendliche in der gleichen Richtlinie geregelt. Insofern gibt es eigentlich keine „Erwachsenen-SAPV“ im Kontrast zu einer „SAPV-KJ“. In Hessen wird die SAPV-KJ allerdings durch pädiatrisch geschulte Teams erbracht. Zur Unterscheidung von Teams, die vor allem erwachsene Patient*innen versorgen, und Teams, die vor allem Kinder und Jugendliche versorgen, benutzen wir die Begriffe Erwachsenen-SAPV und SAPV-KJ.
